# Public assessment of green infrastructure benefits and associated influencing factors in two Ethiopian cities: Bahir Dar and Hawassa

**DOI:** 10.1186/s12898-019-0232-1

**Published:** 2019-04-23

**Authors:** Kassahun Gashu, Tegegne Gebre-Egziabher

**Affiliations:** 1 Department of Geography and Environmental Studies, University of Gondar, P.O. Box 196, Gondar, Ethiopia; 2 Department of Geography and Environmental Studies, Addis Ababa University, P.O. Box 1176, Addis Ababa, Ethiopia

**Keywords:** Benefits, Pearson’s Chi-square test, Green infrastructure, Likert scale, Urban development

## Abstract

**Background:**

Currently, urban green infrastructure is increasingly gaining attention as a source of multiple benefits. Understanding how city residents perceive the benefits of green infrastructure is critical for urban policy and planning. This paper investigates public assessment of the benefits of green infrastructure and the associated influencing factors in Bahir Dar and Hawassa cities of Ethiopia.

**Result:**

Data were collected from residents of the two cities and inferential and descriptive statistics were used to identify public assessment of benefits of green infrastructure and the factors that influence their assessment of benefits of green infrastructure. Findings revealed that people either agree or strongly agree to the triple benefits (environmental, economic and socio-cultural) of green infrastructure. The Pearson’s Chi-square test results depict that, except a few, most of the demographic, socio-economic and bio-physical factors have no significant influence on environmental, economic and socio-cultural benefits of green infrastructure.

**Conclusion:**

This study implies that an understanding of the public assessment of the benefits of green infrastructure can provide important inputs to promote participatory green infrastructure planning.

## Background

At present cities are undergoing substantial transformations following economic and demographic changes and the urbanization process. As a result, cities face complex environmental problems such as pollution, loss of biodiversity, overpopulation, and land consumption [1]. Green infrastructure is a strategic planning approach that can cope and respond to these challenges through the provision of ecosystem services and the benefits of these services [1].

In terms of planning for green infrastructure, participation of stakeholders has been emphasized by some authors [1, 2] because it helps peoples’ voices to be heard and their requirements to be met in the planning and design process. Different aspects of participation in green infrastructure are discussed in the literature. Previous research [3] discusses who? and how? of stakeholder involvement, while others address when? and how?. Equally important in this regard is to identify how people perceive green infrastructure benefits since this will provide information on their preferences and values regarding green infrastructure [4].

Understanding how city residents perceive the benefits of green infrastructure is critical for urban policy and planning concerned with social justice, equity, well-being and sustainability [5]. This is particularly important in Sub-Saharan Africa where there is a rapid pace of urbanization and need for urban environments should secure meaningful and quality of life [6]. The results could be good pointers for concerned authority to work on co-management of green infrastructure by including peoples’ assessment of green infrastructure in their planning and design endeavors. In addition, it is also important to understand how the different demographic, socio-economic and bio-physical factors influence people’s perception of these benefits. These factors could be used as predictors of peoples’ assessment and help to understand why people perceive the different benefits in a very different manner.

To the best of our knowledge, studies concerning the benefits of green infrastructure and the influencing factors are not well represented in previous studies in Ethiopia. The objective of this study is to examine public assessment of the benefits of green infrastructure in their locality and to identify the associated factors that influence public assessment of the benefits by taking Bahir Dar and Hawassa cities as case studies.

### Benefits of green infrastructure

Nowadays, urban green infrastructure is increasingly gaining attention as a source of multiple benefits [7–13]. Different authors [14–16] defined multi-functionality of green infrastructure as composite functions that include environmental, socio-cultural, and economic benefits. The environmental benefits [17] of green infrastructure include local temperature moderation during hot weather [10, 18], cooling of the air temperature through shade provision [19, 20] and mitigation of urban heat island effects [21, 22]. The roles of green infrastructure in reducing noise pollution [23], in mitigating flood [23, 24] in enriching biodiversity and improving ecosystem [12, 25, 26] are also taken as environmental benefits.

Socio-cultural benefits are the non-material benefits people obtain from green infrastructure [17]. These include educational values, aesthetic values, social relations, sense of place, cultural heritage values, recreation, ecotourism and psychological well-being [27]. Socio-cultural benefits of green infrastructure for urban residents include mental and physical health improvements such as stress reduction and relaxation [28–30]. Further, green infrastructure directly increases the quality of life through active and passive recreational social benefits [31]. Physical activities such as sports, playing with children etc. signify active benefits while the passive ones include relaxations, meeting with friends, or experiencing nature and the like [32]. In addition, green infrastructure acts as meeting place for local residents and facilitates social interaction [33, 34]. These elements illustrate the range and the breadth of socio-cultural benefits [29] of green infrastructure and they cover two specific elements: (1) benefits people derive from their feelings of being connected to green infrastructure and, (2) benefits people derive from diversity and complexity of green infrastructure. Unfortunately, the socio-cultural dimensions of green infrastructure have received much less attention than the environmental considerations in infrastructure planning and development endeavors [35, 36].

Economic benefits [17] of green infrastructure come from the increased values of properties near green spaces and the increased sales of properties along green commercial corridors [37]. In this line, studies in China [38, 39] and Adelaide in Australia [40] indicated that higher housing prices and property values are evident for sites situated in the vicinity of accessible green infrastructure. In addition to this, the economic benefits of green infrastructure include its contributions to tourism [37, 41, 42] and its link with urban food [43, 44]. With regard to the latter, a study by Pitman and Ely [45] explained that green infrastructure and urban food are intimately related through the perceived needs to retain productive agricultural land on the urban fringe and to integrate food production into urban areas.

### Understanding factors influencing public assessment of green infrastructure benefits

As outlined above, green infrastructure provides a range of benefits [17] linked with environmental, economic and socio-cultural benefits, but more specific information is required about the factors which influence peoples’ assessment of these broad benefits. The relationship between benefits of green infrastructure and factors which influence peoples’ assessment of these benefits is based on the concept that one can understand a person’s interaction with his or her physical and socio-cultural surroundings [46]. This section, therefore, tries to scrutinize the major groups of factors that affect peoples’ assessment of the benefits of green infrastructure.

A number of previous studies [10, 47–50] showed that demographic factors such as gender, age, and marital status are likely to influence public assessment of the benefits of green infrastructure. Some authors [47, 51, 52] also depicted that socio-economic factors such as educational status, income and home ownership were apparently some predictors of peoples’ assessment of the benefits of green infrastructure. In addition, bio-physical factors [53] such as types of green infrastructure, size of green infrastructure, distance between home to infrastructure, preferred visit time, duration of visit, safety of green infrastructure, public transportation access to green infrastructure are also thought to have an influence on the peoples’ assessment of benefits of green infrastructure [50, 54, 55]. With regard to bio-physical factors previous studies [56, 57] showed that the frequency of the use of green infrastructure is influenced by distance and size of infrastructure. Moreover, some studies [58] estimated that distance is a better predictor for the perception of the benefits of green infrastructure. A distance of 300–400 m is seen as a typical threshold value after which the frequency of using infrastructure starts to decline [56, 57] for particular type of green infrastructure such as green open space or park. However, it does not necessarily hold true for other types of green infrastructure, for example street trees or green walls.

## Methods

### Study areas

**Fig. 1:**
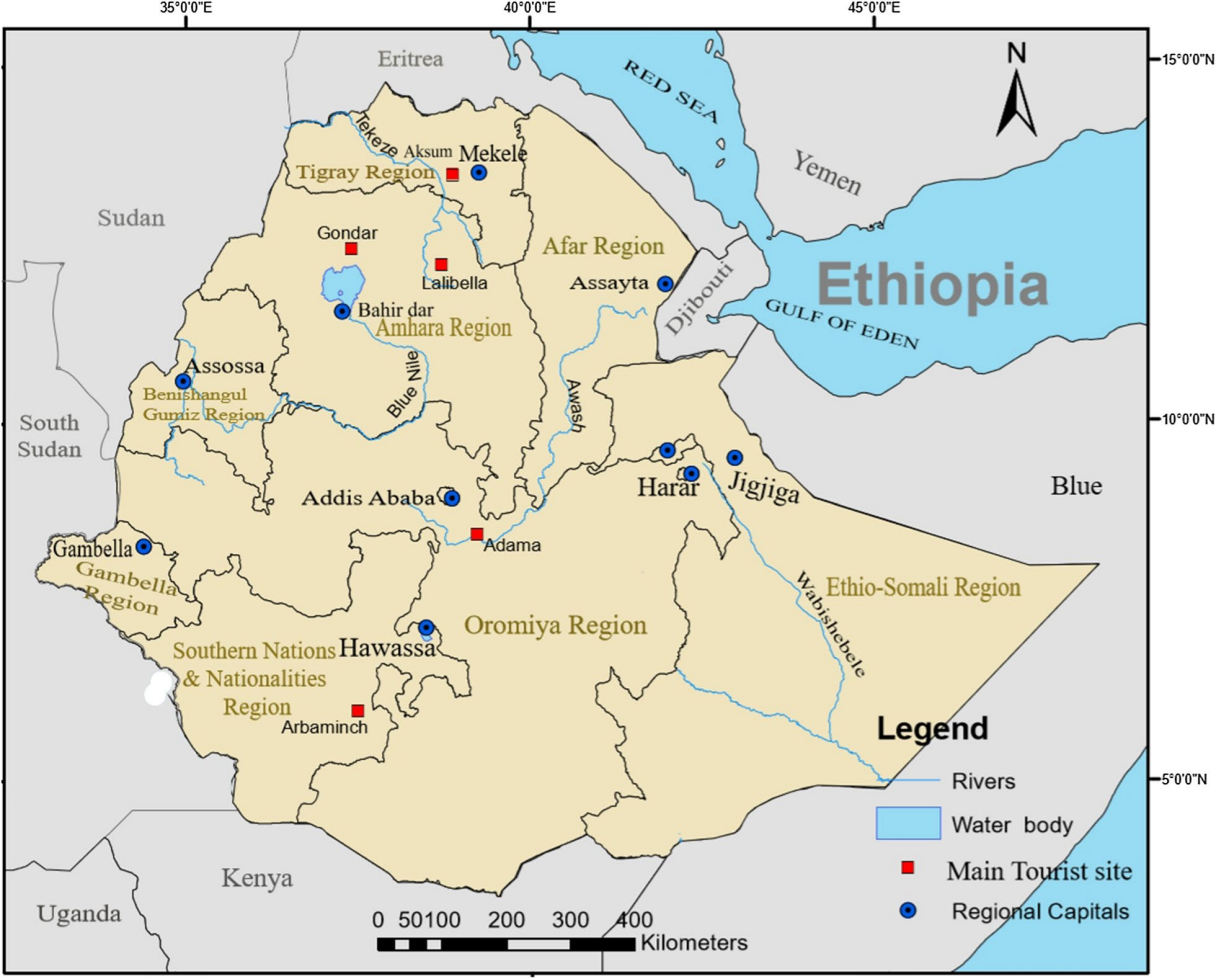
Location of study areas (Source: Own formulation using GIS software application)

The data used in this study were collected in Bahir Dar and Hawassa cities, which are located in Amhara and Southern Nations, Nationalities and Peoples (SNNP) regions respectively (Fig. 1). Bahir Dar is the capital of Amhara region and Hawassa is the capital of Southern Nations, Nationalities Region. Bahir Dar is located at 11°36′North and 37°23′ East and has an average elevation of 1801 m above the sea level and Hawassa is located at 07°03′ North and 38°28′ East. Both cities to a large extent lie on a flat plain.

These two cities are among the largest and fastest growing cities in Ethiopia. The first national population and housing census, conducted in 1984, put the population of Bahir Dar city at 54, 773, while the second national population and housing census, conducted after 10-years, shows that the total population grew to 96,140 [59, 60]. According to the Central Statistical Authority (CSA), the population of the city was projected to be 348,429 in 2017 [61]. In a similar way, the first and second national population and housing censuses put the population of Hawassa to be 36,367 in 1984 and 69,169 in 1994 [59, 60]. The CSA projection of the city population for 2017 was 315,267 [61]. The rapid growths of population in both cities imply their importance in the Ethiopian urban system.

According to the definition given by the Ministry of Urban Development, and Housing (MoUDH) cited in Gashu and Gebre-Egziabher [62] and Gashu et al. [63] adopted green infrastructure typologies to include parks, sports fields, roadside and squares, plazas and festive areas, river and riverside areas, lakes and lakeside areas, watershed areas, urban agriculture development, woodlots and green belts (inside and surrounding forests), private compounds and surroundings, institutional compounds and surroundings (both governmental and non-governmental), communal housing compounds and surroundings (condominiums, real estate, etc.), religious institutions compounds and surroundings, neighborhood open spaces, cemeteries, nursery sites, and green roofs and walls [64]. Based on this, Hawassa has green infrastructure coverage of 21.96% [65] while Bahir Dar city has green infrastructure coverage of 17.44% [66].

### Sample

A two-stage random sampling technique was employed to select the sample respondents in each city. In the first stage, five sub-cities were purposively selected in each city since both have a comparable number of sub-cities (nine in Bahir Dar and eight in Hawassa) and these sub-cities are with relatively better availability of green infrastructure in each city and are close to the lakes in each city (Lake Tana in Bahir Dar and Lake Hawassa in Hawassa). The selected sub-cities in Bahir Dar were Facilo (population size: 26,349), Hidar-11 (population size: 33,950), Shum-abo (population size: 31,221), Geshi-abay (population size: 19,938) and Sefene-selam (population size: 20,236) [67]. The sub-cities selected in Hawassa were Misrak (population size: 30,350), Menaheria (population size: 29,120), Tabor (population size: 25,125), Mehalketema (population size: 24,135) and Haik Dar (population size: 21,201) [68]. In the second stage, a total sample size was determined using a formula [69].

1 $$n_{0} = \frac{{Z^{2} pq}}{{e^{2} }}$$

where n_0_: the sample size; Z: the value found in statistical tables which contain the area under the normal curve that cuts off an area α at the tails (1 − α equals the desired confidence level, e.g., 95%) (1.96); p: the estimated proportion of an attribute that is present in the population (0.5); q: 1 − p; e: the desired level of precision (0.05).

Hence, using the above formula (1), the calculated number of sample size is 384. This number of sample size was also substantiated by Creative Research Systems [70] sample size calculation method. Moreover, published table values also demonstrate 384 samples for 1,000,000 population size [71–73]. The computed sample size which was 384 was augmented to a total of 430 (215 from each city) by an addition of 46 more samples from both cities (23 in Bahir Dar and 23 in Hawassa) in order to increase representation of the sample respondents. In each city, respondents were randomly selected from the five sub-cities based on sub-city roaster used as a sampling frame. The total sample size was distributed to the selected sub-cities based on a proportion to size method to each sub-city. The proportional numbers of respondents in each sub-city were selected using the proportional sampling method. Sample size for each sub-city can be proportionately calculated in the following way:

2 $$ni = \left[ {\frac{Ni}{N}} \right]\;*\;{\text{n}}$$

where, ni is sample size for each sub-city, Ni is population of each sub-city, N is total population of each city and n is total sample size.

### Data analysis and statistical methods

Data were collected using a 5-point Likert scale (Totally agree; Agree; Neutral; Disagree; Totally disagree) to the public assessment of potential benefits of green infrastructure. Descriptive statistical methods such as frequencies, means, and percentages were used to summarize the information on respondents’ responses. To analyze public assessment of the benefits of green infrastructure, we tested systematically for the independence among demographic (gender, age, marital status), socio-economic (educational level, income level, house ownership) and bio-physical (type of green infrastructure, size of green infrastructure, average walking distance from home to green infrastructure, duration of visits to green infrastructure, preferred visit moment, safety, public transport access) factors and benefits of green infrastructure using Pearson’s Chi-square test (χ^2^) of independence. We searched for differences in the distribution of those factors using χ^2^ tests of independence. This test gives consistent results in all cases [4]. For every independence test performed, χ^2^ yield similar conclusions at any given usual level of confidence. We used 5% as our rejection limits. The tests were applied to (r × c) contingency tables with r (rows) and c = 2 columns (i.e. Benefits of green infrastructure vs. one of our three types of factors). Therefore, all calculated statistics follow χ^2^ with (r − 1) × (c − 1) = r − 1 degrees of freedom. We also found it interesting to test whether benefits of green infrastructure were different by type regarding the stipulation (or not) of skills. This leads to (2 × 2) contingency tables with 1 d.f. We applied Yates’ continuity correction for Pearson’s χ^2^ in these cases. All corrected Pearson’s χ^2^ were found consistent with the uncorrected result.

## Results

### Socio-demographic characteristics of sample respondents

The survey distributed 430 questionnaires in the two cities and all respondents returned a completed questionnaire (100% response rate). The questionnaires were distributed equally between Bahir Dar (N = 215, 50%) and Hawassa (N = 215, 50%). Table 1 presents the demographic and socio-economic characteristics of the respondents. In terms of age, almost all respondents in both cities are within the active working age group except a few (0.9%) that are above the working age group (Table 1).

**Table 1:** Socio-demographic characteristics of respondents (N = 215 each city). Source: Survey result

Attributes	Bahir Dar	Hawassa
Gender (%)		
Male	82.3	75.3
Female	17.7	24.7
Age (in years)	µ = 41, δ = 21	µ = 44, δ = 23
18–24	18.1	17.2
25–34	34.9	30.7
35–44	25.6	22.8
45–54	17.7	21.9
55–64	2.8	6.5
64+	0.9	0.9
Marital status (%)		
Married	68.8	64.7
Not married	27.9	25.6
Divorced	3.3	5.6
Widowed	–	4.2
Education (%)		
Grade 1–8	7.9	12.6
Grade 9–12	14.4	18.8
College/university student	8.8	11.6
College/university graduate	68.8	57.2
Family size	µ = 3.6, δ = 2.5	µ = 4, δ = 2
1–3	51.6	40.5
4–6	36.7	44.2
6+	11.6	15.3
Monthly income (Birr)	µ = 2500	µ = 2750
580–2000	42.8	33.5
2001–4000	34.0	40.9
4001–7000	16.7	21.4
> 7000	6.5	4.2
Main income source (%)		
Self employed	33.0	33.5
Private business/NGO employed	23.7	24.2
Government employed	42.8	40.0
Pensioner	0.5	2.3

µ: mean; δ: standard deviation

With regard to education, almost all respondents have attended formal school in both cities. In Bahir Dar, more than 77% of the respondents have attended college/university or have graduated from college/university while the remaining 23% has either completed primary or secondary education (Table 1). In Hawassa, more than 67% of the respondents have attended either college/university or have graduated from college/university. The remaining 33% has either completed primary or secondary education (Table 1). The educational status of the respondents’ indicates that respondents may have a better understanding of green infrastructure.

The average family size for Bahir Dar (3.6) and Hawassa (4.0) is less than the national average (4.6). The majority of the respondents (88%) in Bahir Dar have six or fewer family members and a small proportion (11%) has more than six members of family. Respondents in Hawassa have similar family structure with 85% having six or fewer members and 15% having more than six members. The average monthly income of respondents in Bahir Dar is 2500Birr,^1^ while respondents in Hawassa have a slightly higher average income (2750 Birr). At this juncture, it is important to note that on average about 57% of the respondents got their income from self-employment or private business/NGO employment, while only 41% earn their income from government employment.

### Assessment of green infrastructure benefits and factors influencing benefits

#### Public assessment of green infrastructure benefits

Public assessment of green infrastructure benefits was captured by asking people to rate the different sub-components of the major benefits-Environmental, Economic and Socio-Cultural on a 5-point Likert scale. The Likert scale was structured with lower values (1 and 2) assigned to positive ratings and higher values (4 and 5) assigned to negative ratings and middle value (3) assigned as neutral.

The results presented in Table 2 show that almost all of the respondents have rated positively the benefits by indicating that they either strongly agree or agree to the different sub-components of the benefits. The top three sub-components to which the majority of respondents strongly agree for environmental benefits are temperature moderation (82.3%), air quality improvement (74.9%) and biodiversity conservation (73.5%) in Bahir Dar while they are temperature moderation (77.7%), biodiversity preservation (77.7%) and urban heat island mitigation effect (71.2%) in Hawassa. It thus appears that the effects of green infrastructure on temperature and biodiversity are the one’s highly perceived by the respondents (Table 2). In terms of economic benefits, increase in tax revenue (60%), attracting more customers to the business (53%) and attracting investment or economic activities (52.6%) are the three most important components to which the majority agreed in Bahir Dar while in Hawassa they are residential property (61.4%), food source (60.5%) and, property values (59.5%) (Table 2). This shows that respondents value different components of economic benefits in Hawassa and Bahir Dar. With regard to socio-cultural benefits however, the educational value, psychological wellbeing and relaxation benefits are components to which respondents both in Bahir Dar and Hawassa agreed strongly.

**Table 2:** Public assessment of potential benefits of green infrastructure in Bahir Dar and Hawassa cities (N = 215 for each city). Source: Survey result

Potential benefits	Bahir Dar							Hawassa						
	1 (%)	2 (%)	3 (%)	4 (%)	5 (%)	6	7	1 (%)	2 (%)	3 (%)	4 (%)	5 (%)	6	7
Environmental benefits														
Temperature moderation	82.3	17.7	0.0	0.0	0.0	1.354	0.0398	77.7	21.4	0.5	0.0	0.5	1.404	0.0406
Air quality improvement (pollution control)	74.9	25.1	0.0	0.0	0.0			64.7	34.9	0.5	0.0	0.0		
Noise reduction (sound pollution reduction)	55.3	44.7	0.0	0.0	0.0			48.8	50.2	0.9	0.0	0.0		
Biodiversity conservation	73.5	26.5	0.0	0.0	0.0			77.7	22.3	0.0	0.0	0.0		
Water harvesting	58.1	41.4	0.5	0.0	0.0			44.2	52.6	3.3	0.0	0.0		
Water quality improvement	55.8	43.7	0.5	0.0	0.0			46.0	52.6	1.4	0.0	0.0		
Flood protection	64.2	35.8	0.0	0.0	0.0			46.5	50.2	2.8	.5	0.0		
Urban heat island mitigation effect	70.7	29.3	0.0	0.0	0.0			71.2	27.0	0.9	.9	0.0		
Man–environment ecological relationship improvement	61.9	37.7	0.5	0.0	0.0			70.7	27.9	0.9	.5	0.0		
Rural–urban linkage improvement	52.1	47.4	0.5	0.0	0.0			65.5	33.5	1.4	0.0	0.0		
Economic benefits														
Property values	43.7	54.0	1.9	.5	0.0	1.556	0.038	38.6	59.5	1.9	0.0	0.0	1. 630	0.0264
Food source	48.4	50.7	0.5	.5	0.0			37.7	60.5	1.9	0.0	0.0		
Commercial vitality	47.4	51.6	0.9	0.0	0.0			39.1	58.6	2.3	0.0	0.0		
Residential property	48.8	50.7	0.5	0.0	0.0			36.7	61.4	1.9	0.0	0.0		
Promoting investment or economic activity	47.0	52.6	0.5	0.0	0.0			39.5	59.1	1.4	0.0	0.0		
Increase in tax revenue	38.6	60.0	1.4	0.0	0.0			39.1	57.7	2.8	.5	0.0		
Attracting more customers to the business/tourism	45.6	53.0	1.4	0.0	0.0			45.1	52.1	2.3	0.0	0.5		
Socio cultural benefits														
Educational value	75.8	23.7	0.5	0.0	0.0	1.494	0.0285	71.6	28.4	0.0	0.0	0.0	1. 476	0.0324
Play spaces	58.6	41.4	0.0	0.0	0.0			41.4	57.2	1.4	0.0	0.0		
Psychological wellbeing	64.7	35.3	0.0	0.0	0.0			63.3	36.3	0.0	0.0	.5		
Attractive living spaces	56.7	42.8	0.5	0.0	0.0			56.3	42.8	.9	0.0	0.0		
Lifespan increase	36.7	61.9	1.4	0.0	0.0			32.6	64.2	2.8	0.0	0.5		
Social interaction	39.5	59.1	0.9	.5	0.0			49.8	48.4	1.4	0.0	0.5		
Human physical wellbeing	43.3	55.3	1.4	0.0	0.0			48.4	48.8	2.8	0.0	0.0		
Human social wellbeing	41.9	57.2	0.5	0.0	0.5			56.3	42.3	1.4	0.0	0.0		
Recreation/relaxation	60.9	38.6	0.5	0.0	0.0			67.0	32.1	0.5	0.0	0.5		
Sense of safety for residents	37.7	60.9	1.4	0.0	0.0			55.3	43.3	1.4	0.0	0.0		

1: totally agree; 2: agree; 3: neutral; 4: disagree; 5: totally disagree; 6: aggregated mean; 7: standard deviation

The aggregate measures show that peoples’ perception of both the environmental and the economic benefits are stronger in Bahir Dar than in Hawassa while the socio-cultural benefits (1.4) are rated similar in both cities. People’s perception of aggregated environmental benefit in Bahir Dar is 1.3 while respondents in Hawassa rated it as 1.40. Aggregate economic benefit is rated as 1.5 in Bahir Dar and 1.6 in Hawassa (Table 2).

#### Factors influencing benefits of green infrastructure

The triple benefits of green infrastructure are widely acknowledged by respondents in both cities as the majority of them agree to the presence of these benefits in their cities. In all cases, two-third or more of the respondents responded positively to the different types of benefits. The majority (77%) in Hawassa acknowledged environmental benefits while the majority (75%) in Bahir Dar acknowledged economic benefits. In both cities, social benefits of green infrastructure are acknowledged by lesser proportion (68% in Hawassa and 66% in Bahir Dar). This is surprising because one may assume that the biodiversity of green infrastructure can be immediately used for social and recreational purposes while both environmental and economic benefits are long term benefits. The less attention given to the social benefits is also evident in the literature (see above). The details of results for better understanding of the factors that influence assessment of benefits of green infrastructure are presented in the following sub-sections.

#### Demographic factors

As indicated above the demographic factors specified are gender, age and marital status. The **χ**^**2**^ tests of independence result for Hawassa and Bahir Dar show that there is no statistically significant association (p > 0.05) between gender and environmental benefit, between gender and economic benefits and between gender and socio-cultural benefits (Table 3). In other words both males and females equally understood that green infrastructure deliver environmental, economic and socio cultural benefits both in Bahir Dar and Hawassa.

**Table 3:** Chi-square value of demographic factors and benefits of green infrastructure in Hawassa and Bahir Dar (N = 215 each city). Source: Survey result

Variables	Hawassa								Bahir Dar							
	EnvB		EcoB		SocB		Total		EnvB		EcoB		SocB		Total	
	Yes	No	Yes	No	Yes	No			Yes	No	Yes	No	Yes	No		
	N	N	N	N	N	N	N	%	N	N	N	N	N	N	N	%
Demographic factors																
Gender																
Male	128	34	118	44	110	52	162	75.3	125	52	135	42	116	61	177	82.33
Female	37	16	39	14	36	17	53	24.7	29	9	27	11	29	9	38	17.67
Total	165	50	157	58	146	69	215	100	154	61	162	53	145	70	215	100
χ^2^ and (df = 1)	χ^2^ = 1.89, p = 0.169		χ^2^ = 0.11. p = 0.91		χ^2^ = 0.00, p = 0.99				χ^2^ = 0.499, p = 0.48		χ^2^ = 0.46, p = 0.49		χ^2^ = 1.66, p = 0.19			
Age																
18–24 years	32	6	28	10	22	16	38	17.6	54	16	54	16	56	14	70	32.56
25–44 years	84	32	82	34	78	38	116	54.0	62	37	72	27	64	35	99	46.05
45–64 years	64	12	47	14	46	15	61	28.4	38	8	36	10	25	21	46	21.39
Total	165	50	157	58	146	69	215	100	154	61	162	53	145	70	215	100
χ^2^ and (df = 2)	χ^2^ = 2.86, p = 0.241		χ^2^ = 0.83, p = 0.66		χ^2^ = 3.35, p = 0.12				χ^2^ = 7.72, p = 0.02*		χ^2^ = 0.69, p = 0.71		χ^2^ = 8.97, p = 0.01*			
Marital status																
Married	124	35	116	43	110	49	159	74.0	110	38	112	36	96	52	148	68.84
Not married	27	8	24	11	23	12	35	16.2	39	21	45	15	42	18	60	27.91
Widowed/divorced	14	7	17	4	13	8	21	9.8	5	2	5	2	7	0	7	3.25
Total	165	50	157	58	146	69	215	100	154	61	162	53	145	79	215	100
χ^2^ and (df = 2)	χ^2^ = 1.34, p = 0.513		χ^2^ = 1.02, p = 0.60		χ^2^ = 0.54, p = 0.762				χ^2^ = 1.83, p = 0.403		χ^2^ = 0.07, p = 0.96		χ^2^ = 4.0, p = 0.14			

*EnvB* environmental benefit, *EcoB* economical benefit, *SocB* socio-cultural benefit

*p < 0.05

The **χ**^**2**^ tests of independence between age category and different benefits (environmental, economic and socio-cultural) for Hawassa shows that there is no association or there is no significant difference among the age categories (p > 0.05) (Table 3). The **χ**^**2**^ result for Bahir Dar however shows that age category makes difference with respect to environmental benefits (p < 0.05) and socio-cultural benefits (p < 0.05) though it did not make difference with regard to economic benefits (p > 0.05). In Bahir Dar, those in the age group 25–44 have formed the majority of those who felt that green infrastructure has environmental and socio-cultural benefits.

The marital status of respondents i.e., whether the respondents are married, divorced/widowed, or not married does not have any statistical association with the perception of the different benefits in both Hawassa and Bahir Dar. In all cases the **χ**^**2**^ result revealed no association (p > 0.05) (Table 3).

#### Socio-economic factors

The socio-economic factors are those pertaining to education, income levels, and house tenure. The education variable differentiates between those with elementary, high school and university/college graduates. The **χ**^**2**^ result for the association between education and green infrastructure benefits show that there is no significant difference (p > 0.05) between and among the education level in both Hawassa and Bahir Dar in perceiving the green infrastructure benefits (Table 4). This result is surprising because one may think that as individuals’ attain higher education, their perception of the infrastructural benefits will be different from those with lower level of education.

**Table 4:** Chi-square value of socio-economic factors and benefits of green infrastructure in Hawassa and Bahir Dar (N = 215 each city). Source: Survey result

Variables	Hawassa								Bahir Dar							
	EnvB		EcoB		SocB		Total		EnvB		EcoB		SocB		Total	
	Yes	No	Yes	No	Yes	No			Yes	No	Yes	No	Yes	No		
	N	N	N	N	N	N	N	%	N	N	N	N	N	N	N	%
Socio-economic factors																
Education status	20	7	20	7	20	7	27	12.6	12	5	13	4	13	4	17	7.91
Elementary	28	12	29	11	27	13	40	18.6	22	9	23	8	20	11	31	14.42
High school	117	31	108	40	99	49	148	68.8	120	47	126	41	112	55	167	77.67
College/university graduated	165	50	157	58	146	69	215	100	154	61	162	53	145	70	215	100
χ^2^ and (df = 2)	χ^2^ = 1.57, p = 0.46		χ^2^ = 0.21, p = 0.99		χ^2^ = 0.544, p = 0.76				χ^2^ = 0.02, p = 0.99		χ^2^ = 0.034, p = 0.98		χ^2^ = 0.76, p = 0.68			
Monthly income	53	19	47	25	44	28	72	33.5	69	23	70	22	68	24	92	42.79
580–2000Birr	72	17	70	19	64	25	89	41.4	46	27	56	17	50	23	73	33.95
2001–4000Birr	33	12	35	10	30	15	45	20.9	29	7	28	8	18	18	36	16.74
4001–7000Birr	7	2	5	4	8	1	9	4.2	10	4	8	6	9	5	14	6.51
> 7000Birr	165	50	157	58	146	69	215	100	154	61	162	53	145	70	215	100
χ^2^ and (df = 3)	χ^2^ = 1.56, p = 0.67		χ^2^ = 5.53, p = 0.14		χ^2^ = 4.03, p = 0.26				χ^2^ = 4.6, p = 0.204		χ^2^ = 2.71, p = 0.45		χ^2^ = 6.84, p = 0.08			
House tenure	90	29	86	33	82	37	119	55.3	90	33	92	31	81	42	123	57.21
Own house	75	21	71	25	64	32	96	44.7	64	28	70	22	64	28	92	42.79
Rented	165	50	157	58	146	69	215	100	154	61	162	53	145	70	215	100
Total	90	29	86	33	82	37	119	55.3	90	33	92	31	81	42	123	57.21
χ^2^ and (df = 1)	χ^2^ = 0.185, p = 0.67		χ^2^ = 0.77, p = 0.78		χ^2^ = 0.122, p = 0.73				χ^2^ = 0.34, p = 0.56		χ^2^ = 0.05, p = 0.83		χ^2^ = 0.33, p = 0.57			

*EnvB* environmental benefit, *EcoB* economical benefit, *SocB* socio-cultural benefit

*p < 0.05

The **χ**^**2**^ result for income levels indicate that there is no statistical difference (p > 0.05) between income levels and green infrastructure benefit. In other words those people with different income levels perceive similarly the green infrastructure benefits in their city. House tenure in the form of home ownership and rental status also makes no difference (p > 0.05) with regard to the perception of green infrastructure benefits (Table 4).

#### Bio-physical factors

The last category of factors are the bio-physical factors which include type of green infrastructure, size of green infrastructure, average walking distance from home to green infrastructure, duration of visit, preferred visit moment, safety of infrastructure and access to public transport.

As presented in Table 5, the **χ**^**2**^ results show that there is no difference among respondents regarding their perception of the different types of benefits and type of green infrastructure visited. The only exception is among respondents who identified socio-cultural benefits in Bahir Dar city. In Bahir Dar, it appeared that the respondents’ perception of socio-cultural benefits has an association with type of infrastructure visited (p < 0.05) (Table 5). This is because, in this city, those who visited road medians and lake side view are the majority to perceive socio-cultural benefits of infrastructure.

**Table 5:** Chi-square value of bio-physical factors and benefits of green infrastructure in Hawassa and Bahir Dar (N = 215 each city). Source: Survey result

Variables	Hawassa								Bahir Dar							
	EnvB		EcoB		SocB		Total		EnVB		EcoB		SocB		Total	
	Yes	No	Yes	No	Yes	No	N	%	Yes	No	Yes	No	Yes	No	N	%
	N	N	N	N	N	N			N	N	N	N	N	N		
Type of green infrastructure																
Public parks and open spaces	48	12	42	18	39	21	60	27.6	28	15	32	11	33	10	43	20.0
Squares, plaza festival sites and sport fields	38	12	31	19	31	19	50	23.3	42	13	41	14	40	15	55	25.58
Road medians and lake side view	63	20	65	18	61	22	83	38.6	74	30	78	26	60	44	104	48.37
Others not mentioned	16	6	19	3	15	7	22	10.2	10	3	11	2	12	1	13	6.05
Total	165	50	157	58	146	69	215	100	154	61	162	53	145	70	215	100
χ^2^(df = 3)	χ^2^ = 1.41, p = 0.09		χ^2^ = 2.12, p = 0.05		χ^2^ = 1.3, p = 0.05				χ^2^ = 1.69, p = 0.06		χ^2^ = 0.65, p = 0.09		χ^2^ = 10.56, p = 0.01*			
Size of green infrastructure^a^																
Small	43	10	38	15	34	19	53	24.7	28	11	35	4	24	15	39	18.14
Medium	77	28	81	24	70	35	105	48.8	106	42	104	44	100	48	148	68.84
Large	45	12	38	19	42	15	57	26.5	20	8	23	5	21	7	28	13.02
Total	165	50	157	58	146	69	215	100	154	61	162	53	145	70	215	100
χ^2^(df = 2)	χ^2^ = 1.41, p = 0.09		χ^2^ = 2.12, p = 0.05		χ^2^ = 1.3, p = 0.53				χ^2^ = 0.001, p = 0.07		χ^2^ = 7.10, p = 0.03*		χ^2^ = 1.35, p = 0.51			
Average distance of green infrastructure from home (walking distance)																
< 10 min	61	21	59	23	55	27	82	38.1	22	7	18	11	18	11	29	13.49
Between 10 and 30 min	68	20	63	25	59	29	88	40.9	67	29	75	21	62	34	96	44.65
> 30 min	36	9	35	10	32	13	45	21.0	65	25	69	21	65	25	90	41.86
Total	165	50	157	58	146	69	215	100	154	61	162	53	145	70	215	100
χ^2^(df = 2)	χ^2^ = 0.54, p = 0.76		χ^2^ = 0.66, p = 0.72		χ^2^ = 0.27, p = 0.87				χ^2^ = 0.43, p = 0.81		χ^2^ = 3.24, p = 0.198		χ^2^ = 1.67, p = 0.43			
Preferred visit moment																
Evening	86	31	87	5	77	40	20	9.3	76	30	82	24	73	33	106	49.30
Afternoon	64	14	55	23	55	23	78	36.3	72	31	75	28	68	35	103	47.91
Morning	15	5	15	30	14	6	117	54.4	6	0	5	1	4	2	6	2.79
Total	165	50	157	58	146	69	215	100	154	61	162	53	145	70	215	100
χ^2^(df = 2)	χ^2^ = 1.95, p = 0.34		χ^2^ = 0.34, p = 0.82		χ^2^ = 0.52, p = 0.77				χ^2^ = 2.53, p = 0.28		χ^2^ = 0.79, p = 0.67		χ^2^ = 0.19, p = 0.91			
Waiting time while visiting green infrastructure																
< 1 h	65	21	63	23	55	31	86	40.0	81	31	84	28	77	35	112	52.09
Between 1 and 2 h	96	29	91	34	88	37	125	58.1	68	29	74	23	63	34	97	45.12
> 2 h	4	0	3	1	3	1	4	1.9	5	1	4	2	5	1	6	2.79
Total	165	50	157	58	146	69	215	100	154	61	162	53	145	70	215	100
χ^2^(df = 2)	χ^2^ = 1.28, p = 0.53		χ^2^ = 0.013, p = 0.99		χ^2^ = 1.07, p = 0.587				χ^2^ = 2.53, p = 0.28		χ^2^ = 0.79, p = 0.67		χ^2^ = 0.19, p = 0.91			
Safety of green infrastructure																
Safe	145	46	136	55	135	56	191	88.8	140	56	147	49	135	61	196	91.16
Not safe	20	4	21	3	11	13	24	11.2	14	5	15	4	10	9	19	8.84
Total	165	50	157	58	146	69	215	100	154	61	162	53	145	70	215	100
χ^2^(df = 1)	χ^2^ = 0.66, p = 0.02*		χ^2^ = 2.87, p = 0.03*		χ^2^ = 6.04, p = 0.01*				χ^2^ = 0.04, p = 0.04*		χ^2^ = 0.15, p = 0.01*		χ^2^ = 2.08, p = 0.05			
Public transportation access to visit green infrastructures with reasonable cost																
Accessible	106	29	94	41	85	50	135	62.8	134	41	131	44	119	56	175	81.40
Not accessible	59	21	63	17	61	19	80	37.8	20	20	31	9	26	14	40	18.60
Total	165	50	157	58	146	69	215	100	154	61	162	53	145	70	215	100
χ^2^(df = 1)	χ^2^ = 0.64, p = 0.02*		χ^2^ = 2.12, p = 0.04*		χ^2^ = 4.07, p = 0.04*				χ^2^ = 11.31, p = 0.001*		χ^2^ = 0.12, p = 0.03*		χ^2^ = 0.133, p = 0.02*			
Status of green infrastructure visited																
Fair	34	14	37	11	30	18	48	22.5	28	4	23	9	20	12	32	14.88
Good	47	13	45	15	43	17	60	27.9	70	34	77	27	70	34	104	48.37
Very good	66	17	59	24	56	27	83	38.6	43	16	45	14	39	20	59	27.44
Excellent	18	6	16	8	17	7	24	11.2	13	7	17	3	16	4	20	9.31
Total	165	50	157	58	146	69	215	100	154	61	162	53	145	70	215	100
χ^2^(df = 3)	χ^2^ = 1.42, p = 0.05		χ^2^ = 1.17, p = 0.06		χ^2^ = 1.14, p = 0.77				χ^2^ = 5.4, p = 0.05		χ^2^ = 1.33, p = 0.07		χ^2^ = 1.84, p = 0.01*			

*df* degree of freedom, *EnvB* environmental benefit, *EcoB* economical benefit, *SocB* socio-cultural benefit

*p < 0.05

^a^In view of the predominance of the relatively small-sized holdings less than or equal to 200 m^2^ were labeled small, those between 200 and 500 m^2^ were labeled medium, and those more than 500 m^2^ were labeled large [73]

The size of green infrastructure refers to whether the green infrastructure visited is small, large or medium in its size. The **χ**^**2**^ results indicate that there are no associations (p > 0.05) between the perception of respondents of all types of benefits in both Bahir Dar and Hawassa and the size of green infrastructure visited. In Bahir Dar city, however, those who perceived green infrastructure to have economic benefits showed association with the size of green infrastructure visited (p < 0.05) (Table 5). In other words those who visited medium sized green infrastructure are the majority to perceive green infrastructure to have economic benefits.

The average walking distance between green infrastructure site and one’s home (measured in minutes), the preferred time for visit and the duration of visit showed no association with the respondents’ perception of the different green infrastructure benefits in both Bahir Dar and Hawassa cities (p > 0.05) (Table 5).

The safety of green infrastructure visited is an important bio-physical factor. The **χ**^**2**^ results show that respondents’ perception indicated association with safety in Hawassa and not in Bahir Dar. In Hawassa those who felt that the green infrastructure visited is safe are also the majority to perceive that green infrastructure to have both economic and socio-cultural benefits (p < 0.05) (Table 5). The result for accessibility of green infrastructure to public transport showed association with respondents’ perception of social benefits in Hawassa city and environmental benefits in Bahir Dar city.

## Discussion

This paper sought to examine public assessment of the benefits of green infrastructure and the factors that influence these benefits in Bahir Dar and Hawassa cities of Ethiopia. The recognition of urban residents’ assessment of the urban green infrastructure benefits would certainly optimize green infrastructure planning processes. This study is a starting step towards a better understanding of how urban residents’ rate the benefits associated with urban green infrastructure and it should have great importance for policy and practice. Our findings revealed that respondents do recognize and appreciate the multiple benefits of green infrastructure in their respective city. Almost all of the respondents either strongly agree or agree to multiple benefits of green infrastructure in both cities.

Among the components of environmental benefit, the one that is stated by most of the respondents in both cities is temperature moderation [51, 74–76]. Temperature moderation is valued highly since higher temperature results in discomfort that is felt immediately both at work places and at home. Similarly, among the components of the socio-cultural benefits, educational value and psychological wellbeing are cited by many people. This is because, in many developed and developing countries, green infrastructure can be used as training sites for education and they can also be used for recreation sites for psychological wellbeing. However, there is variation in the perception of benefits of green infrastructure in the other types and components of green infrastructure benefits. This variation is may be due to administration difference, geographical variation, and cultural differences in the two cities. The result on the benefits matches with the studies conducted by Tyrvainen et al. in Helsinki [77], by Lo and Jim in Hong Kong [78] and by Jim and Shan in Guangzhou [79], in which green infrastructure contribution to multi-functional benefits is recognized. In addition, a study by Peckham et al. in two Canadian cities [80] indicated that access to nature affects urban citizens’ physical and mental well-being

The result on the factors influencing people’s benefit depicted that there is no significant association between most of the demographic factors and the environmental, economic and socio-cultural benefits. One of the implications of this is that gender and marital status do not create difference in the perception of benefit by people. In other words, males and females understand the benefits of green infrastructure similarly as do people with different marital status [10, 81]. Age is among the demographic factors that showed significant relation with socio-cultural benefit, but like other demographic factors it too has no significant association with other types of benefits both in Bahir Dar and Hawassa.

The result also indicated that socio-economic factors do not make difference in people’s assessment of the different types of benefits. The result is in line with some studies in the literature [7, 51, 82]. The bio-physical factors also showed no relationship with the perception of benefits. The only exception in this regard is that the size of green infrastructure creates difference in the perception of socio-cultural and economic benefits in Bahir Dar. Larger size is thus expected to induce higher socio-cultural and economic benefits.

In Hawassa, safety of green infrastructure has significant influence on public assessment of socio-cultural benefit. This implies that safer green infrastructure generated higher social benefits. Public transport is also found to have significant influence on socio-cultural and environmental benefits in Hawassa and Bahir Dar. Thus those green infrastructure sites that are easily accessible to public transport provide better social and environmental benefits for the people. A study in Turkey [83] depicted that green infrastructure network should be elaborated beautifully and extended to more areas. Additionally, good public transportation system and enough parking lots could attract more beneficiaries far away from the urban green infrastructure.

Relatively bio-physical factors influence public assessment of benefits of green infrastructure than demographic and socio-economic factors. This is in line with some previous studies [81, 84, 85]. Bio-physical factors thus need to be considered in determining peoples’ value and preferences regarding the benefits of green infrastructure [86, 87].

## Conclusion

Urban green infrastructure strategies can be developed by understanding how urban residents benefits from it [88]. As described earlier, since these (environmental, economic and socio-cultural) benefits of green infrastructure are main pillars of sustainable development, the main challenge to urban planning and decision-making is therefore to accommodate a diversity of desirable social, environmental, and economic green infrastructure benefits [88].

This study provides insights on the assessment of the benefits of green infrastructure and factors influencing the assessment of the benefits. The result showed that most people agree to the existence of different benefits and their components. In terms of factors, though most of the factors in this study have no significant influence on public assessment of benefits of green infrastructure, it is instructive to take note of the bio-physical factors.

Urban planners who seek to promote participatory green infrastructure planning and design need to be aware of the different sub components of green infrastructure highly valued by people and the specific factors that make difference in people’s assessment of the green infrastructure benefits. Hence, joining these triple benefits into an integrated scheme of green infrastructure helps for urban planners, managers and the society at large to think about urban sustainable development.

## Abbreviations

C: columns
CSA: Central Statistical Agency
d.f.: degree of freedom
EcoB: economic benefit
EnvB: environmental benefit
GIS: Geographic Information System
ha: hectares
m: meter
µ: mean
δ: standard deviation
MoUDH: Ministry of Urban Development, and Housing
N: number
NGO: non-governmental organizations
r: rows
SNNP: Southern Nations, Nationalities and Peoples
SocB: socio-cultural benefit
χ^2^: Pearson’s Chi-square test

## Footnotes

1 1US Dollar was exchanged with 27.57 Birr in the time of data collection.
